# Mitochondrial quality control alterations and placenta-related disorders

**DOI:** 10.3389/fphys.2024.1344951

**Published:** 2024-02-08

**Authors:** Yamei Wu, Meng Li, Hao Ying, Ying Gu, Yunlong Zhu, Yanfang Gu, Lu Huang

**Affiliations:** ^1^ Wuxi Maternity and Child Healthcare Hospital, Affiliated Women’s Hospital of Jiangnan University, Wuxi, China; ^2^ Wuxi Clinical Medical College of Nanjing Medical University, Wuxi, China; ^3^ Shanghai First Maternity and Infant Hospital, Tongji University School of Medicine, Shanghai, China

**Keywords:** mitochondrial dysfunction, preeclampsia, foetal growth restriction, placenta, mitochondria

## Abstract

Mitochondria are ubiquitous in eukaryotic cells. Normal maintenance of function is the premise and basis for various physiological activities. Mitochondrial dysfunction is commonly observed in a wide range of pathological conditions, such as neurodegenerative, metabolic, cardiovascular, and various diseases related to foetal growth and development. The placenta is a highly energy-dependent organ that acts as an intermediary between the mother and foetus and functions to maintain foetal growth and development. Recent studies have demonstrated that mitochondrial dysfunction is associated with placental disorders. Defects in mitochondrial quality control mechanisms may lead to preeclampsia and foetal growth restriction. In this review, we address the quality control mechanisms of mitochondria and the relevant pathologies of mitochondrial dysfunction in placenta-related diseases, such as preeclampsia and foetal growth restriction. This review also investigates the relation between mitochondrial dysfunction and placental disorders.

## 1 Introduction

Mitochondria are double-membrane organelles consisting of dynamic inner and outer membranes. Mitochondria are the main sites of biological oxidation and energy conversion in eukaryotic cells. Mitochondria are involved in the regulation of several physiological mechanisms. These include energy metabolism (Zhang et al., 2021), biosynthesis (Ryoo and Kwak, 2018; Fuchs et al., 2020), calcium signal transduction (Jouaville et al., 1998), production of reactive oxygen species (ROS) (Hernansanz-Agustin and Enriquez, 2021; Kuznetsov et al., 2022), and apoptosis (Wang et al., 2021; Tian et al., 2022). Therefore, mitochondrial dysfunction may lead to various diseases, such as cardiovascular diseases, skeletal muscle disorders, and neurodegenerative diseases (Mani et al., 2021; Sadoshima et al., 2021; Chen et al., 2022; Vodickova et al., 2022). Mitochondrial homeostasis is maintained at the organelle level through dynamically regulated quality-control mechanisms, including mitochondrial fusion and fission, biogenesis, and mitophagy (Green and Van Houten, 2011; Kotiadis et al., 2014; Suliman and Piantadosi, 2016). At the molecular level, homeostasis is mediated by the mitochondrial unfolded protein response, mitochondrial molecular chaperones, and proteases which constitute the mitochondrial protein quality control system that maintains the dynamic balance of the mitochondrial proteome (Vazquez-Calvo et al., 2020).

The placenta consists of the amniotic membrane, chorionic villous membrane, and decidua basalis, which are important organs for maintaining foetal growth and development. Therefore, it has a high energy demand. The placenta performs material exchange, defence, synthesis, and immunity (Shao et al., 2022). Mitochondria consume oxygen via oxidative phosphorylation to produce adenosine triphosphate (ATP). When mitochondrial dysfunction occurs, ATP synthesis is reduced and placental function is impaired, resulting in various pregnancy complications, such as preeclampsia (Marin et al., 2020; Smith et al., 2021) and foetal growth restriction (Kiyokoba et al., 2022). Furthermore, mitochondrial dysfunction amplifies oxidative stress and the production of ROS, leading to a self-perpetuating cycle of mitochondrial damage. This, in turn, contributes to placental dysfunction (Manna et al., 2019). In this study, we explored the intricate relation between mitochondrial dysfunction and placenta-related diseases, focusing on their role in conditions, such as preeclampsia and foetal growth restriction.

## 2 Mitochondrial quality control mechanism

### 2.1 Mitochondrial fusion and fission

The morphology, size, and number of mitochondria are controlled by constant fusion and fission (Zemirli et al., 2018). In mammalian cells, mitochondrial outer membrane (MOM) fusion is mainly regulated by the dynamin-related GTPases mitofusin 1 and mitofusin 2 (MFN2) (Sloat et al., 2019), whereas mitochondrial inner membrane fusion is mainly regulated by optic atrophy 1, which is also a dynamin-related GTPase (Youle and van der Bliek, 2012). Moreover, Misato protein (MSTO1) is a soluble cytoplasmic protein. MSTO1 translocates to the MOM and interacts with mitochondrial fusion proteins at the MOM–cytoplasm interface (Al Ojaimi et al., 2022). F-Box and Leucine-rich repeat protein 4 is a nuclear-encoded mitochondrial protein that is in the intermembrane space. F-Box and Leucine-rich repeat protein 4 may also play a role in mitochondrial fusion by interacting with and regulating mitochondrial fusion proteins (Wang et al., 2020). Proteins involved in mediating mitochondrial fission include dynamin-related protein 1 (Drp1), fission protein 1, and mitochondrial dynamic proteins MiD49 and MiD51 (Serasinghe and Chipuk, 2017). The phosphorylation of Drp1 at Ser637 controls the mitochondrial fission-promoting activity of Drp1 (Kanamaru et al., 2012). cAMP-dependent protein kinase phosphorylates Drp1 and inhibits its translocation to mitochondrial fission sites. In contrast, calcineurin-mediated dephosphorylation of Drp1 causes Drp1 to accumulate in the mitochondria and promotes mitochondrial fission (Suarez-Rivero et al., 2016). Mitochondrial dynamics are complex and are related to mitochondrial function through fusion and fission. Research has indicated that mitochondrial dynamics play a crucial role in various pathological manifestations, including neurodegenerative diseases, cancer, cardiomyopathy, and metabolic disorders (Serasinghe and Chipuk, 2017).

### 2.2 Mitophagy

**FIGURE 1:**
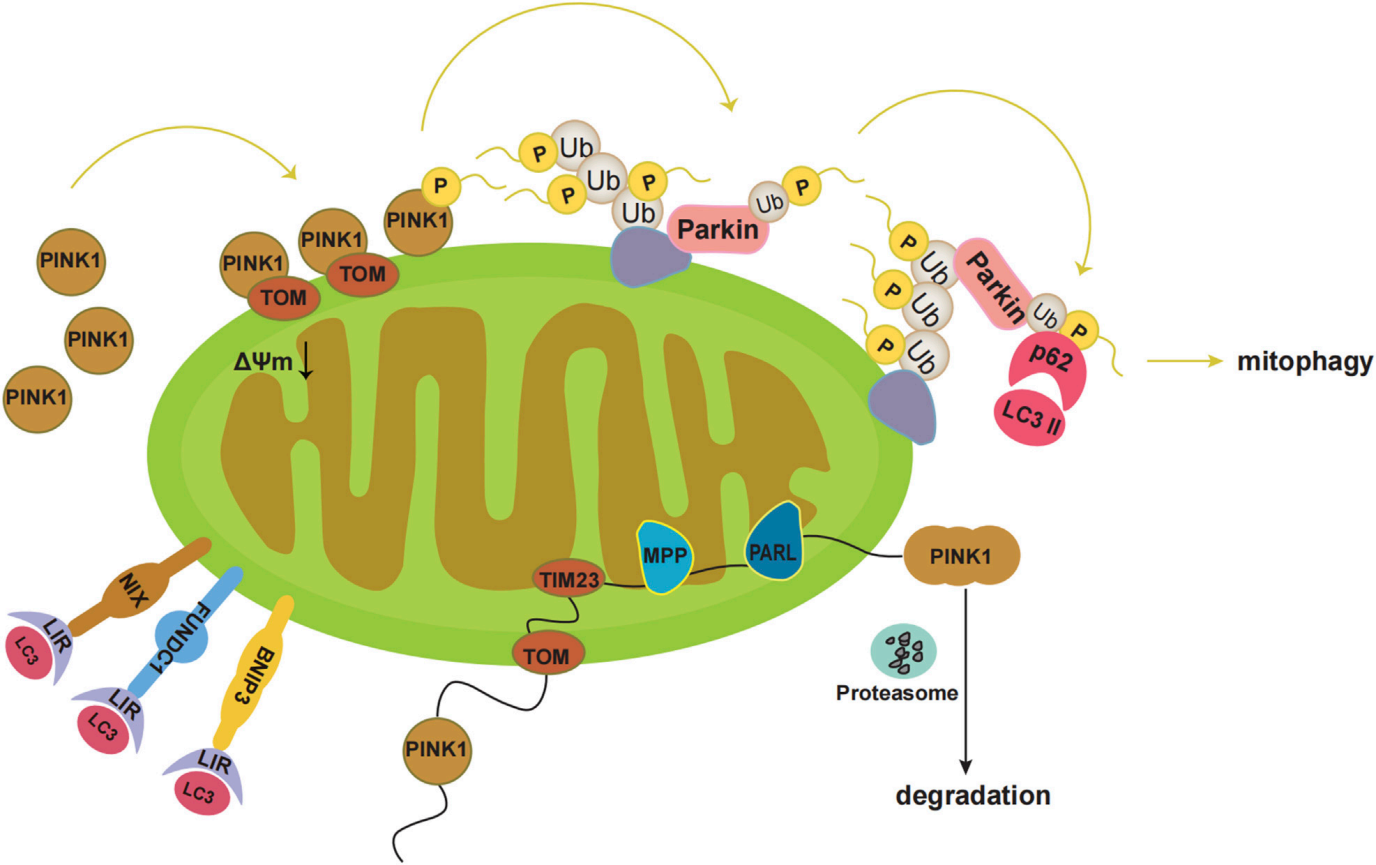
Pathways of mitophagy. Under physiological conditions, PINK1 is localized to the mitochondrial outer membrane, which is introduced into the intermembrane space by translocase outer membrane (TOM). Then PINK1 is introduced into the mitochondrial inner membrane by translocase of the inner membrane 23 (TIM23). At the mitochondrial inner membrane, the mitochondrial processing peptidase (MPP) removes PINK1’s N-terminal MTS. Subsequently, PGAM5-associated rhomboid-like protease (PARL) cleaves PINK1. Finally, PINK1 in the double-cleavage form is released into the cytoplasm and is degraded rapidly by the ubiquitin proteasome system. Under pathological conditions, the damaged mitochondria undergo mitochondrial membrane depolarization. PINK1 accumulates at the mitochondrial outer membrane. Then PINK1 kinase is activated by autophosphorylation, which directly phosphorylates Ser65 in the parkin Ubl domain, or first phosphorylates Ser65 in ubiquitin and leads to conformational changes in parkin, then causes the release of the Ubl domain from the parkin core, and finally phosphorylates Ser65 in the parkin Ubl domain. Subsequently, p-Parkin ubiquitinates mitochondrial outer membrane proteins, which bind sequestosome1 (p62) and microtubule-associated protein 1 light chain 3 II (LC3II). Eventually mitophagy occurs. Additionally, BNIP3, NIX, and FUNDC1 can interact with the autophagosome membrane protein LC3 through the LC3 interaction region (LIR).

Mitophagy refers to the process by which cells selectively remove aging and damaged mitochondria through autophagy, thereby controlling mitochondrial quality and maintaining mitochondrial homeostasis (Tian et al., 2022). There are two main mitophagy pathways, the ubiquitin-mediated and the receptor-mediated pathway (Chen et al., 2022). The ubiquitin-mediated pathway includes the PINK1-Parkin pathway. PINK1 is localised to the MOM and is introduced into the intermembrane space by the translocase outer membrane and into the mitochondrial inner membrane by the translocase of the inner membrane 23. At the mitochondrial inner membrane, PINK1 is processed with mitochondrial processing peptidase and PGAM5-associated rhomboid-like protease, and eventually degraded (Figure 1). Under pathological conditions, damaged mitochondria undergo mitochondrial membrane depolarisation. In the PINK1-Parkin pathway, when the mitochondria are damaged and undergo membrane depolarisation, PINK1 accumulates in the MOM. PINK1 is then activated through autophosphorylation, leading to the direct or indirect phosphorylation of Ser65 in the parkin Ubl domain. Subsequently, p-parkin ubiquitinates mitochondrial outer membrane proteins, forming complexes with sequestosome1 (p62) and microtubule-associated protein 1 light chain 3 II. These events ultimately result in mitophagy (Figure 1) (Eldeeb et al., 2022; Wu et al., 2022; Yildirim et al., 2022).

In addition, various mitochondrial proteins, such as NIP-3-like protein X (NIX), FUN14 domain-containing 1 (FUNDC1), and BNIP3 can act as mitophagy receptors to guide the clearance of damaged mitochondria (Xie et al., 2020; Linqing et al., 2021; Xie et al., 2021; Liu et al., 2022; Ning et al., 2022). BNIP3, NIX, and FUNDC1 interact with the autophagosomal membrane protein LC3 via the LC3 interaction region (Figure 1) (Wei et al., 2015). Phosphorylation of Ser34/35 near the LC3 interaction region of NIX enhances its binding to LC3 (Rogov et al., 2017). In addition, NIX may induce mitophagy by promoting the production of ROS (Li et al., 2015; Li et al., 2022). Both NIX and BNIP3 are considerably upregulated by HIF-1α under hypoxic conditions, facilitating the clearance of damaged mitochondria and preventing ROS accumulation (Mazure and Pouyssegur, 2010). NIX may act as a substrate for Parkin in the PINK1-Parkin pathway (Gao et al., 2015). BNIP3 may interact with PINK1 to prevent its proteolytic activity and support its accumulation on the mitochondrial membrane, thereby promoting mitophagy (Zhang et al., 2016). FUNDC1 mediated mitophagy is primarily regulated through phosphorylation and ubiquitination. In the presence of oxygen, FUNDC1 is phosphorylated by Src kinase at Tyr18 and by CK2 at Ser13 (Zhang et al., 2022). Under hypoxic conditions, FUNDC1 is ubiquitinated by lysine 119 of the mitochondrial E3 ligase membrane-associated RING-CH5, which protects mitochondria from degradation by mitophagy. During severe hypoxia, CK2 and Src kinases are inhibited, dephosphorylating FUNDC1 and increasing its binding affinity to LC3, thereby promoting mitophagy (Chen et al., 2017a).

## 3 Mitochondrial protein quantity control system

Mitochondria have a bilayer membrane structure with approximately 140 proteins in the outer membrane, 130 soluble proteins in the intermembrane space, and 800 and 500 proteins in the inner membrane and matrix, respectively (Jadiya and Tomar, 2020). Molecular chaperones, proteases, the ubiquitin-proteasome system, mitophagy, mitochondria-derived vesicles, and the mitochondrial unfolded protein response are involved in mitochondrial protein quality control (Friedlander et al., 2021; Song et al., 2021).

Molecular chaperones, such as Hsp60 (Fan et al., 2020) and Hsp70 (Havalova et al., 2021), can promote and maintain the correct folding of proteins to ensure their normal function. Mitochondrial proteases play crucial roles in controlling the quantity of mitochondrial proteins. They are responsible for protein turnover and processing within mitochondria (Steele and Glynn, 2019; Szczepanowska and Trifunovic, 2022). The ubiquitin-proteasome system is involved in the degradation of more than 80% of endogenous proteins in eukaryotes, and ubiquitin molecules ubiquitinate and degrade substrate proteins mainly through the formation of multiubiquitin chains (Alsayyah et al., 2020; Kodron et al., 2021). Currently, the specific mechanism by which mitochondria-derived vesicles are involved in mitochondrial protein quality control is not clear. Several studies have pointed out that vesicles contain damaged MOMs, inner membranes, and matrix proteins that are transported to lysosomes for degradation (Soubannier et al., 2012), suggesting that vesicles may be a complementary mechanism that controls mitochondrial quality prior to mitochondrial degradation at the organelle level (Baixauli et al., 2014). The mitochondrial unfolded protein response is a stress response. Under stress signalling conditions, such as decreased mitochondrial membrane potential, mitochondrial DNA (mtDNA) clearance, mitochondrial accumulation of misfolded proteins, or an imbalance between nuclear and mitochondrial-encoding proteins, the gene transcription of nuclear-encoded mitochondrial chaperones and proteases is activated. This activation enhances protein homeostasis (Svagusa et al., 2020). Tight-coupled molecular interactions between multiple players in the mitochondrial protein quality control system ensures protein balance and mitochondrial function to maintain overall cellular fitness.

## 4 Placenta-related diseases

### 4.1 Preeclampsia

Preeclampsia is a multisystem disorder that occurs during pregnancy, characterised by new-onset hypertension (systolic pressure/diastolic pressure, ≥140/90 mmHg) and proteinuria (≥300 mg/24 h) after 20 weeks of gestation. In 2013, the American Conference of Obstetricians and Gynecologists noted that proteinuria was not necessary for the diagnosis of preeclampsia. Preeclampsia can also be diagnosed as hypertension associated with thrombocytopenia, renal insufficiency, liver impairment, pulmonary oedema, or brain or visual impairment.

The association between mitochondrial dysfunction and preeclampsia was first explored in 1989, and several members of a family with mitochondrial dysfunction were found to have a high incidence of preeclampsia and eclampsia (Torbergsen et al., 1989). This was followed by a steady stream of studies linking mitochondrial dysfunction to preeclampsia. One study reported morphological data showing degeneration and apoptotic changes in the mitochondria of the preeclamptic placenta (Shi et al., 2013). The study also found that compared with those in normal placentas, preeclampsia placentas had four upregulated mitochondrial proteins and 22 downregulated mitochondrial proteins that are involved in many key processes in the development of preeclampsia, such as apoptosis, fatty acid oxidation, respiratory chain, ROS generation, tricarboxylic acid cycle, and oxidative stress. Thus, mitochondrial dysfunction is closely associated with the occurrence and development.

As research has progressed in recent years, our understanding of the specific mechanisms underlying preeclampsia has deepened. It has become increasingly clear that mitochondrial dysfunction plays a central role in the molecular mechanism of preeclampsia, particularly when it is caused by dysfunction in placental 11β-hydroxysteroid dehydrogenase type 2 (Long et al., 2022). Their study revealed that dysfunction in 11β-hydroxysteroid dehydrogenase type 2 resulted in mtDNA instability and impaired mitochondrial dynamics, contributing to the development of preeclampsia, and the mitochondrial-targeted antioxidant MitoTEMPO has significantly alleviated the symptoms of preeclampsia.

Interestingly, conflicting conclusions exist in the literature regarding the expression of mitochondrial fusion and fission proteins in preeclamptic placentas. These discrepancies in protein expression may be attributed to variations in the severity of preeclampsia and differences in study populations. Fusion is a process that helps preserve mitochondrial function by mixing the contents of damaged mitochondria, whereas fission is the first step in mitophagy. The mitochondrial fusion proteins mitofusin 1, MFN2, and optic atrophy 1 are downregulated in the placentas of women with preeclampsia (Zhou et al., 2017). Another study also showed that MFN2 and ATP expression was significantly reduced in the preeclamptic placenta compared with that in normal placenta (Yu et al., 2016). However, maternal serum MFN2 levels were higher in patients with preeclampsia (Aydogan Mathyk et al., 2020). In conclusion, despite conflicting findings from various studies, it is evident that there is a disruption in the balance between mitochondrial fusion and fission in the preeclamptic placenta.

Mitophagy serves the function of eliminating damaged mitochondria. Defects in the autophagic pathway may contribute to the onset and progression of preeclampsia. A previous study found that the inhibition of BNIP3 expression reduced autophagic activity, leading to the accumulation of damaged mitochondria *in vivo*, thus participating in the development of preeclampsia (Zhou et al., 2021). The downregulation of BNIP3 expression in preeclamptic placentae has also been confirmed in other studies (Tong et al., 2018; Ma et al., 2019). These results suggest that decreased autophagy is closely associated with preeclampsia. However, another study found contradictory results. Low levels of FUNDC1 ubiquitination have been found in hypoxic trophoblast cells and the placentas of pregnant women with preeclampsia (Chen et al., 2022). Mitochondrial E3 ligase membrane-associated RING-CH5 has been reported to inhibit hypoxia-induced mitochondrial autophagy through ubiquitination and degradation of FUNDC1, and inhibition of FUNDC1 ubiquitination and degradation increases mitochondrial sensitivity to autophagy-induced stress (Chen et al., 2017a; Chen et al., 2017b). Therefore, FUNDC1 ubiquitination is a process of mitochondrial desensitisation that prevents functional mitochondria from being removed via autophagy. The decrease in FUNDC1 ubiquitination promotes autophagy, resulting in excessive enhancement of autophagy and destruction of normal mitochondria, which are related to the occurrence and development of preeclampsia. In summary, changes in autophagic activity are associated with preeclampsia.

### 4.2 Foetal growth restriction

Foetal growth restriction is defined as foetal body mass or abdominal circumference less than the 10th percentile of body mass for gestational age and is also known as intrauterine growth restriction (IUGR). According to the 2021 guidelines issued by the American College of Obstetricians and Gynecologists, the aetiology of FGR can be divided into maternal, foetal, and placental factors.

Many recent studies have explored the relation between mitochondrial dysfunction and foetal growth restriction. Excessive oxidative stress and perturbed mitochondrial antioxidant capacity can interfere with mtDNA replication, leading to a decrease in mtDNA content in the peripheral blood (Knez et al., 2017). A study found that the mtDNA copy number (mtDNAcn) in the peripheral blood of women with preeclampsia associated with IUGR was significantly lower than that in women with preeclampsia associated with appropriate for gestational age intrauterine foetal growth (Busnelli et al., 2019). Moreover, several studies have reported a reduction in mtDNA copy number levels in the placenta of patients with IUGR (Diaz et al., 2014; Mando et al., 2014; Poidatz et al., 2015; Luo et al., 2018). However, another study found that an increase in mtDNA copy number was an indicator of mitochondrial functional impairment, and the mtDNA content in the IUGR pregnancy group was significantly increased (Naha et al., 2020). Therefore, we speculate that the change in mtDNA copy number is closely related to foetal growth restriction. It was also found that mitochondrial Sirtuin-3 (Sirt3) protein levels were significantly decreased, and succinate dehydrogenase activity was decreased in the IUGR pregnancy group (Naha et al., 2020). Sirt3 is translated as a 44 kDa inactivated protein that is cleaved by 142 amino acids to produce an active 28 kDa protein that acts as a deacetylase on several mitochondrial maintenance proteins (Sundaresan et al., 2008). In addition, mitochondrial Sirt3 has been shown to be associated with ATP synthase subunits to regulate ATP synthesis and maintain membrane potential (Yang et al., 2016). Therefore, decreased Sirt3 protein expression and succinate dehydrogenase activity suggest that mitochondrial function is impaired in growth-restricted placentas.

## 5 Discussion and outlook

The placenta is crucial for the growth and development of the foetus, and because of its high energy demand, the maintenance of mitochondrial function is a necessary condition for the growth and development of the foetus. Studies have shown that abnormalities in mitochondrial fusion and fission, mitophagy, and various mechanisms in the mitochondrial protein quantity control system may lead to mitochondrial dysfunction and placenta-related diseases. Recent studies have shown that exogenous supplementation with antioxidants, such as vitamin D, coenzyme Q10, and melatonin, can improve mitochondrial function, inhibit oxidative stress, reduce inflammatory responses, and protect mitochondria from oxidative damage (Reiter et al., 2020; Phillips et al., 2022). One study has shown that mitochondrial transfer can improve embryo quality and resolve pregnancy complications caused by abnormal mtDNA (Morimoto et al., 2023). However, the specific mechanisms by which mitochondrial dysfunction leads to placental disorders remain unclear. It is expected that the specific molecular mechanisms will be further clarified using animal models and human placental samples to provide new ideas for the prevention and treatment of placenta-related diseases.
